# A New Conversation between Radiology and Pathology-Identifying Microvascular Architecture in Stages of Cirrhosis via Diffraction Enhanced Imaging *In Vitro*

**DOI:** 10.1371/journal.pone.0087957

**Published:** 2014-02-04

**Authors:** Dou-dou Hu, Yu Chen, Ali Bihi, Xin-min Li, Tai-ling Wang, Bao-en Wang, Xin-yan Zhao

**Affiliations:** 1 Liver Research Center, Beijing Friendship Hospital, Capital Medical University, Beijing, China; 2 Institute of High Energy Physics, Chinese Academy of Sciences, Beijing, China; 3 International School of Capital Medical University. Beijing, China; 4 Department of Pathology, China-Japan Friendship Hospital, Beijing, China; University of Campinas, Brazil

## Abstract

**Background/Aim:**

Diffraction enhanced imaging (DEI) is a synchrotron radiation X-ray phase-contrast imaging technique that can better reveal the microstructure of biological soft tissues than conventional X-rays. The aim of this study is to investigate the angio-architectural changes of the liver during fibrosis, cirrhosis and its subsequent regression by applying synchrotron radiation based DEI.

**Methods:**

DEI experiments were performed at the 4W1A station of Beijing Synchrotron Radiation Facility. Twenty-four Sprague-Dawley rats were induced with liver fibrosis by carbon tetrachloride (CCl_4_) for up to 10 weeks, after which spontaneous regression started and continued until week 30. Quantitative analysis of the DEI images yielded the mean vascular density and intercapillary distance, which was then re-confirmed by immunohistochemical analysis of CD34.

**Results:**

Based on the DEI results, the mean vascular density was 1.4-fold higher in fibrotic rats (at week 6) and 2-fold higher in cirrhotic rats (at week 10) compared with the control (*p*<0.05). Accordingly, the intercapillary distance decreased to 563.89±243.35 µm in fibrotic rats and 392.90±92.68 µm in cirrhotic rats compared with 673.85±214.16µm in the control (*p*<0.05). During fibrosis regression at week 30, vascular density was 0.7-fold lower and intercapillary distance increased to 548.60±210.94 µm as compared with cirrhotic rats (*p*<0.05).In parallel to the DEI results, immunohistochemical analysis of CD34 showed similar changes.

**Conclusion:**

Synchrotron-based DEI can conduct radiological as well as pathological analysis. Our results are consistent with previous reports indicating that angiogenesis is directly proportional to fibrosis progression. Furthermore, by clarifying the vascular characteristics of liver diseases, DEI reveals that cirrhosis cannot fully reverse during fibrosis regression.

## Introduction

Cirrhosis is currently ranked as the tenth leading cause of death in the Western World with a 10-year mortality of 34%–66% [1]. The golden sign pertaining to the pathology of cirrhosis includes fibrotic bands, regenerative nodules and vascular distortion that changes normal liver parenchyma [2]. In the past decades, researchers have mainly focused on collagen metabolism as well as antifibrotic therapies in order to inhibit the progression of disease and improve clinical outcomes. Besides collagen accumulation, cirrhosis also involves the formation of new vessels (angiogenesis) and the establishment of abnormal angio-architecture of the liver; this phenomenon has recently gained wider attention in the field of hepatology [3]–[5]. The vascular changes are chiefly attributed to microvascular changes within the intrahepatic circulation. These changes are regarded as one of the most important and newly discovered pathophysiological features of cirrhosis, and contribute to developing and maintaining portal hypertension as well as hindering the reversibility of cirrhosis [6], [7].

Medical imaging techniques such as CT and MRI provide direct anatomical information of hepatic vessels, assisting doctors in decision making, yet the resolution of these techniques is too low to visualize small branches of the portal vein and/or the hepatic vein (< 200μm) [8]. Histopathology of liver tissues (mainly obtained by liver biopsy) is currently regarded as the gold standard for observing subtle vascular structures. However, traditional pathology can only show a given cross section of the microvascular architecture, often yielding discontinuous and incomplete information.

Therefore, it is imperative to develop tools that can detect the angio-architecture of the liver over a wider length scale, from “macro” down to “micro”. We propose the use of synchrotron radiation-based DEI to better understand the changes in intrahepatic vessels during cirrhosis. The DEI set-up is an analyzer-based imaging technique that uses monochromatic and collimated synchrotron radiation beams together with an analyzer crystal placed between the sample and the detector, possessing high spatial resolution and high contrast resolution [9], [10]. As a powerful tool, DEI provides not only the absorption contrast on which conventional imaging techniques rely, but also additional data on soft tissues that were previously accessible only by histopathology [11]. Although DEI has been used to study vascular changes in normal livers [12], [13], it has not been used to study cirrhotic livers in a systemic manner. We believe that DEI has broader applicability in understanding pathological vascular changes during progression and regression of liver cirrhosis.

The aim of this study is to investigate changes in the microvasculature of liver tissues from CCl_4_-induced rats during fibrosis, cirrhosis and regression stages using DEI to quantify the intrahepatic vascular remodeling in different periods of liver cirrhosis. Our study presents an illustrative view of microvasculature changes and an in-depth understanding of the pathogenesis of cirrhosis with regard to the angio-architecture. We postulate that DEI is a new radio-pathological method for assessing the severity of cirrhosis.

## Materials and Methods

### Animal Models

Twenty-four male adult Sprague-Dawley rats, weighing 180–220 g, were maintained in an environmentally controlled room (23±2°C, 55±10% humidity) with a 12-hour light/dark cycle and free accessed to food and water. Liver fibrosis was induced via administration of 2ml of CCl_4_/olive oil (4:6, v/v) per kg body weight by intraperitoneal (i.p.) injection, twice per week for up to 10weeks. The twenty-four rats were euthanized in groups of six at different time intervals, i.e. at weeks 0 (control), 6, 10, and after spontaneous recovery at week 30. The livers from all animals were removed after euthanasia in accordance with the guiding principles for the care and use of laboratory animals approved by the Research Ethics Committee of the Beijing Friendship Hospital, Capital Medical University, China (Permit Number: 12-1004). Liver samples were fixed in neutral phosphate-buffered formalin (10%) and stored at room temperature (25±2°C) and relative humidity of 55±10% until histopathological and DEI analysis.

### Experimental Setup

**Figure 1:**
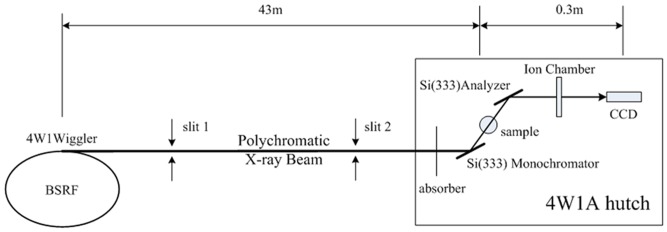
Experimental setup of Diffraction Enhanced Imaging (DEI). Schematic illustration of (DEI) experimental setup at the 4W1A Station in the Beijing Synchrotron Radiation Facility (BSRF).

DEI experiments were performed at the 4W1A station of Beijing Synchrotron Radiation Facility (BSRF). A schematic of the experimental setup is shown in Fig. 1. The X-ray source is a wiggler that emits radiation over a wide energy range (5–20 keV), which is placed at a distance of 43 m from the sample. The size of the incident beam spot on the sample was approximately 20 mm (horizontal) ×11 mm (vertical). The electron energy in the storage ring was 2.5 GeV with a current of 150–250 mA. The white X-ray beam from the 4W1 wiggler was monochromatized by a perfect silicon [333] crystal. After passing through the sample, the X-rays were refracted by an analyzer crystal, identical to the monochromator crystal, and were detected by an X-ray CCD with a pixel size of 7.5 µm×7.5 µm. The spatial resolution of the images is about 30 microns. An aluminum absorber was used to attenuate X-rays of low energy. The DEI experiments were carried out by using the Si [333] diffraction plane of the two crystals to diffract 15 keV X-rays.

### Principle of DEI

**Figure 2:**
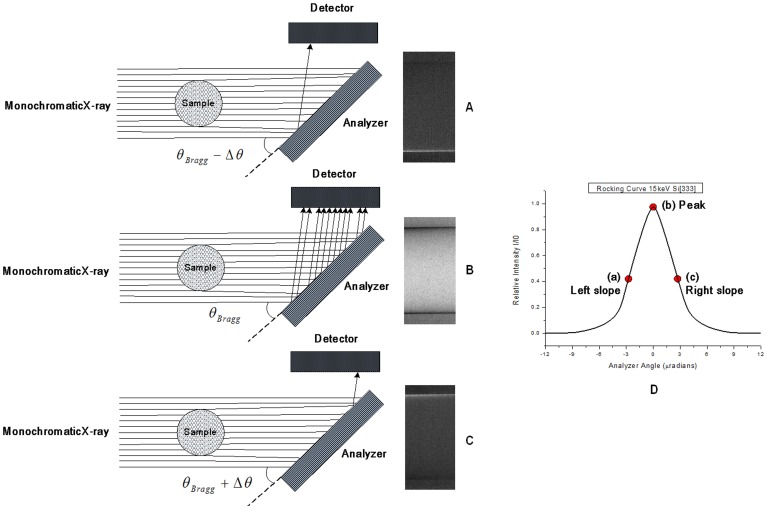
The analyzer crystal selects specifically X-rays which transmitted in a certain angle range incidence on the detector to produce high contrast images (A-C) relative to the position on the RC in (D). A schematic of RC with 15 keV X-rays and silicon [333] diffraction plane (D).

The white X-ray beam from the 4W1 wiggler was made monochromatic using a perfect silicon [333] crystal. After passing through the sample, the intensity of the monochromatic X-ray beam was reduced owing to absorption. Moreover, the incoming beam can be scattered and refracted by the sample through large angles, on the order of degrees, and through small angles, on the order of microradians. The analyzer crystal used in DEI was identical to the monochromator crystal. The silicon crystal has a very narrow angle acceptance range, on the order of microradians called Darwin width (W_D_), which is related to the energy of the X-rays and the diffraction plane of the crystal. Only those photons deviating less than one W_D_ after passing through the sample can be reflected by the analyzer and detected by the detector (Fig. 2A-C).

The angular acceptance range of the analyzer is determined by the width of the rocking curve (RC), i.e. the intensity curve obtained by rocking the analyzer crystal around the Bragg angle with no sample in the beam (Fig. 2D). For the X-ray energy and crystal reflection used here, the width of this curve was a few microradians. Different kinds of images with enhanced contrast can be obtained at different positions on the RC.

### Image Acquisition and Post-processing

**Figure 3:**
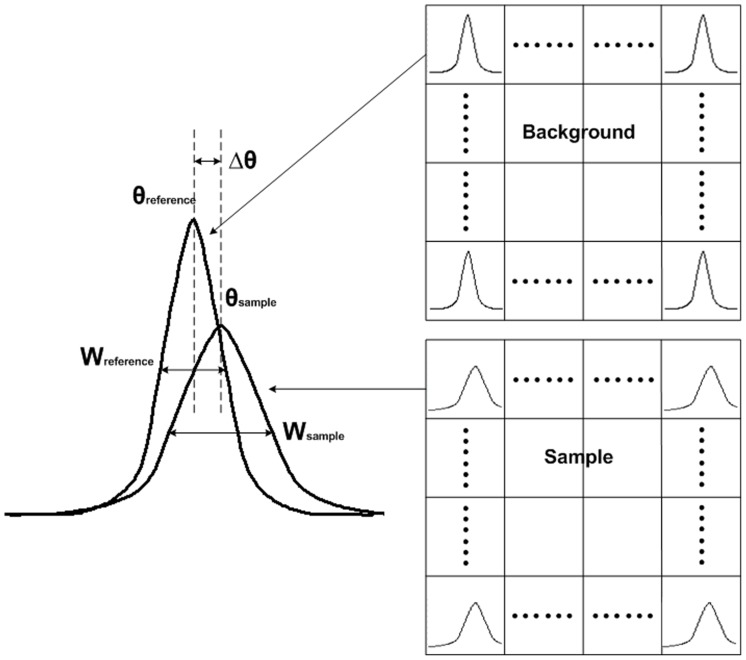
For every pixel, a sample RC and a reference RC can be obtained. The change of the peak, width, area interrelated to the refraction, scattering, absorption of the sample.

Before DEI image acquisition, each liver sample was dehydrated for one to two hours by natural volatilization (23±2°C, 45±10% humidity) to obtain high quality images. Samples were set between two crystals, and a series of images were acquired when the analyzer crystal was set at different positions of the rocking curve of the system. The background images were obtained in the same way, with no sample present in the beam. Thus, two series of images were acquired, one with and one without sample. Each pixel yielded a sample RC and a reference RC. The center of the peak was calculated from both, resulting in a refraction angle image Δθ  =  θ_sample_– θ_reference_. The width of the RC, which was related to the scattering image W =  (W^2^_sample_–W^2^_referencee_)^0.5^, can also be obtained (Fig. 3). The integral of the RC will give the absorption image of the sample. Therefore, refraction, absorption and extinction images of the sample were simultaneously obtained by multiple-image radiography [14]. In general, refraction images have the best contrast; hence, we focus on this type of image in this paper.

### Image Evaluation and Quantification

The mean vascular density was calculated in normal, fibrotic, cirrhotic and regressive livers from our animal models. First, in the direct method, the numbers of microvessels per mm^2^ were independently counted in 10 randomly selected 1 mm^2^ areas per liver sample by two researchers (X-y Zhao and D-d Hu), who were blinded to the stages of cirrhosis, and the mean numbers were calculated. Moreover, we calculated the intercapillary distance under surface edge of the liver in 10 non-overlapping areas to re-confirm the microvascular density, which was adopted from published method [15]. Results were expressed by mean ± SD.

### Observation of Angiogenesis via Immunohistochemistry

Changes in microvasculature were evaluated by immunohistochemical analysis of CD34. Goat polyclonal antibodies against rat CD34 (LS-C150289, LifeSpan BioSciences, Seattle, WA, USA) was used according to the supplier’s instructions. Slides were scanned at low magnification (100×) to identify areas with the greatest vascular density. Subsequently, the microvascular density (MVD) was determined by two pathologists, independently and in masked trials, on 10 non-overlapping areas at 200× magnification. Only immune-positive clusters of cells with a lumen were considered as individual vessels for the purposes of microvessel counting, consistent with the procedures described by Romanenko [16]. Vascular density was expressed as

the mean number of CD34-positive microvessels per 0.785 mm^2^.

### Quantification of Fibrosis

Areas of fibrosis were stained by Sirius red and assessed in five non-overlapping areas at 100× magnification. The fibrosis area was analyzed using Image-Pro Plus 6.0 software (Media Cybernetics, Bethesda, Maryland, USA). The quantity of fibrosis was expressed as a relative percentage between the area of fibrosis and the total area.

### Statistical Analysis

Error bars represented the mean ± SD and were analyzed by the nonparametric ANOVA test, using the software program SPSS ver. 17.0 (IBM, Chicago, IL, USA). All reported *p* values are two-sided, and values with *p*<0.05 were considered statistically significant.

## Results

### Liver Fibrosis Progression and Regression in CCl_4_-treated Rats

**Figure 4:**
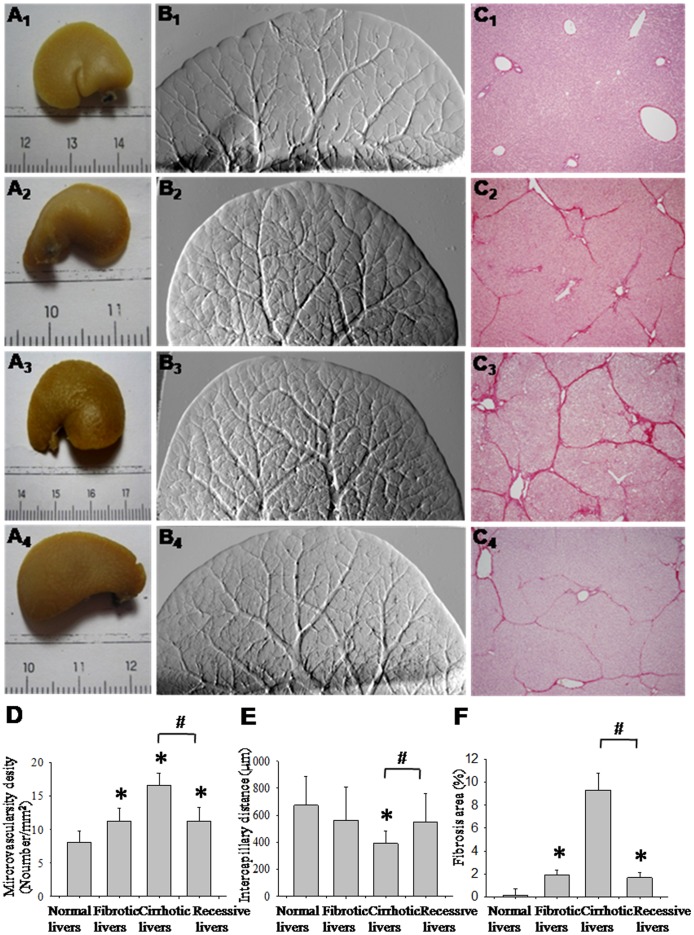
Illustration and quantification of microvasculature and fibrosis in corresponding stages of cirrhosis. Macroscopic observations of liver lobe (A_1-4_) from representative control, fibrotic, cirrhotic and regressive rats, with corresponding DEI vasculature images (B_1-4_) and fibrosis stained by Sirius-Red (C_1-4_). The vasculature of control (B_1_) became more dense at week 6 (B_2_), further increasing in number at week 10 (B_3_). After regression for 30 weeks, the number of micro-vessels decreased (B_4_). The vascular density (D), intercapillary distance (E) and fibrosis area (F) were summarized and expressed as mean ± SD. *p<0.05 compared with control group. ^#^ *p*<0.05 compared with cirrhotic group.

The progression of liver fibrosis in CCl_4_-treated rats was revealed by Sirius red staining (Fig. 4C_1-4_). In normal livers, collagen was detected in low amounts around portal tracts and hepatic veins (Fig. 4C_1_). After CCl_4_ induction for 6 weeks, collagen septa were detected (Fig. 4C_2_); cirrhosis was established at week 10, following which CCl_4_ injection was stopped (Fig. 4C_3_). At week 30, the septa and collagen were reabsorbed and nodules became enlarged (Fig. 4C_4_). Figure 4F shows our quantitative analysis of fibrosis. Fibrotic and cirrhotic rats exhibited fibrosis areas of 1.92±0.44% and 9.30±1.50%, respectively, both of which were significantly higher than the fibrosis area in the control (0.16±0.5%, *p*<0.05). During cirrhosis regression, the fibrosis area decreased to 1.68±0.44% as compared with cirrhotic rats (*p*<0.05).

### Imaging Vascular Changes via DEI

The morphology and branching of hepatic veins and portal veins in the liver lobe were visualized using DEI. In normal livers, the vascular structure resembled a tree, forming regular dichotomous branches (Fig. 4B_1_). Meanwhile, in CCl_4_-treated rats, irregular and tortuous vasculature was observed after 6 weeks (Fig. 4B_2_). In week 10, the microvascular network presented numerous spikes, giving a “hairy” appearance (Fig. 4B_3_). During cirrhosis regression at week 30, the aberrant vascular density diminished considerably, exhibiting more delicate ramifications, though the final structure did not completely revert to that of the control (Fig. 4B_4_).

To complement the aforementioned qualitative study of morphological changes in general, quantitative analysis of the vascular density was performed. Compared with control rats, the mean vascular density was 1.4-fold higher in fibrotic rats and 2-fold higher in cirrhotic rats (*p*<0.05). The vascular density in cirrhotic rats decreased 0.7-fold (*p*<0.05) after 30 weeks regression (Fig. 4D). The changes in micro- vasculature were also quantified by intercapillary distance, which was inversely correlated with the vascular density [17]. The intercapillary width ranged from 563.89±243.35 µm in fibrotic rats to 392.90±92.68 µm in cirrhotic rats, compared with 673.85±214.16 µm in control rats, re-confirming that cirrhotic models had a higher vascular density. During regression, the intercapillary distance markedly increased (548.60±210.94 µm), but did not return to the normal condition (Fig. 4E).

In order to interpret increased vasculature, we compared the DEI images from CCl_4_ model with bile duct ligation model (BDL, see Figure S1). Portal veins can be distinguished from hepatic veins based on the fact that portal veins are normally accompanied with bile ducts. By analysis of DEI images from these two different cirrhotic models (CCl_4_ Vs BDL) along with their corresponding histopathological sections, we concluded that increased vasculature mainly belonged to portal vein systems as well as arterializations of hepatic sinusoids.

### Identifying Vascular Changes by CD34 Immunolabeling

**Figure 5:**
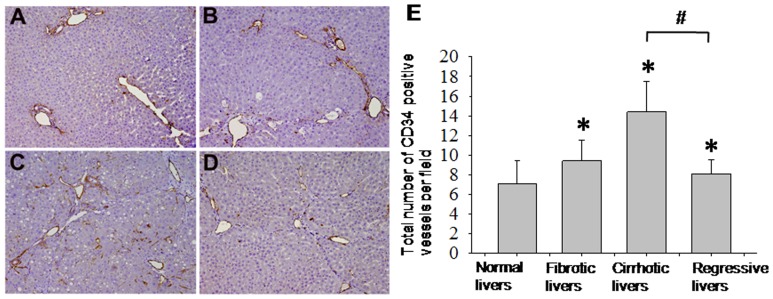
Angiogenesis or neo-vascularization was evaluated by immune- histochemical stain of CD34. CD34 positive microvasculature increases with time; control (A), fibrosis stage at week 6 (B), cirrhosis stage at week 10 (C) and regressive stage after cession of CCl_4_ for 30 weeks (D). Results are expressed as mean ± SD. *p<0.05 compared with the control group. ^#^ *p*<0.05 compared with the cirrhotic group.

To affirm our DEI results regarding the vascular density and morphology, we stained liver tissue sections with an antibody against CD34, an endothelial antigen that is a direct marker for the degree of neoangiogenesis [18] and has been used to highlight vascular density. In normal livers, CD34 expression was restricted to the endothelium of portal veins and hepatic veins (Fig. 5A). In fibrotic livers (6 weeks), numerous CD34-positive vessels were detected in the fibrotic septa (Fig. 5B). In cirrhotic livers (10 weeks), the cirrhotic nodules became surrounded by a dense vascular plexus, and some scattered sinusoidal endothelial cells in these nodules also tested positive for CD34 (Fig. 5C). Conversely, at 30 weeks the number of CD34-labeled vessels decreased and only persisted within the thin fibrous septa (Fig. 5D).

Quantitative analysis of the CD34 staining of endothelial cells revealed an increase of the MVD, to 9.42±2.12 in fibrotic rats and 14.4±3.11 in cirrhotic rats, both of which were statistically significant compared with the MVD of the control group (7.1±2.30, *p*<0.01). After 30 weeks of cirrhosis regression, the MVD was 0.6-fold lower than that of cirrhotic rats (*p*<0.05), as shown in Fig. 5E.

## Discussion

In this study, we explore the use of DEI as a radio-pathological diagnostic tool to study the vascular effects of liver cirrhosis. With the aid of DEI, we first re-confirm previous pathological findings such as the increase in angiogenesis over time during fibrogenesis [3], [19]. Furthermore, during the regression phase of cirrhosis the quantity of blood vessels does in fact decrease but fails to completely return to normalcy [20], [21]. This result corroborates with previous histopathological findings that cirrhosis is not fully reversible especially with regard to the changes in liver microvasculature [20]. The inability of the remodeled vasculature to returning to its original form is possibly due to the complexity of the angio-architecture, and the fundamental changes that it undergoes during the development of cirrhosis [20], [21]. It is worth mentioning that our DEI image results are directly proportional to the results of immunohistochemistry staining of CD34.

More importantly, DEI can potentially be a new radio-pathological method to assess the severity of cirrhosis. Our results indicate that the vascular density of cirrhotic livers is related to the degree of fibrosis, where higher MVD corresponds to more severe cirrhosis. Current methods for determining the severity of cirrhosis and predicting prognosis include the Child-Pugh score and the Model for End-Stage Liver Disease score, both of which rely on functional markers such as albumin, INR, and creatinine [22], instead of evaluating the structural abnormalities corresponding to cirrhosis. Hence, the radio-pathological DEI technique is advantageous for evaluating the severity of cirrhosis by imaging the liver angio-architecture and anatomy; not only does it provide a view of soft tissues, but also a continuous and thorough view. This would complement existing diagnostic tools, allowing clinicians to determine the severity of cirrhosis with greater accuracy.

We selected DEI as a radio-pathological imaging technique because DEI can view the vascular tree in liver lobes, from visible macro-vessels down to small micro-vessels (30 µm) in a non-destructive, continuous and thorough fashion. Compared with traditional image profiles, DEI has the ability to differentiate soft tissues [23], thus achieving in-depth visualization of cirrhotic livers, in particular the microscopic blood vessels. Besides high phase contrast and high spatial resolution, other advantages of DEI include a wider view of the liver; instead of a small specimen as in liver biopsy (1/50,000^th^ of the total liver tissue), it can display a total lobe (approximately 1/9^th^ of the liver tissue) and consequently decrease the likelihood of sampling error. Finally, DEI is easy to perform, time-efficient, and exposes the tissue to a minimal radiation, lower than that of a traditional X-rays [24].

Compared with in vivo microangiography and optical angiography techniques, DEI has the disadvantage of assessing liver samples in vitro. Fresh liver samples are much closer to the in vivo condition than formalin fixed tissues. We initially attempted to perform DEI on fresh liver samples, but DEI was unable to visualize the microvasculature of these samples in the absence of contrast medium. Fresh livers contain fluid inside and outside the vessels, and the difference in electronic density is very small, exceeding the capacity of DEI. After dehydration, formalin volatilized over time, increasing the difference in electronic density and enabling vessels to be detected by DEI. To our knowledge, microangiography is used to assess the hepatic artery systems (i.e. to detect microvascular lesions) rather than the portal or hepatic venous systems by injection of contrast medium into a peripheral artery. Optical microangiography was recently developed to visualize microcirculatory tissue beds in vivo. Since this new technique only can visualize superficial microvessels up to 2.00 mm beneath the tissue surface [25], and since the liver is located deep within the abdominal cavity, optical microangiography has not yet been used to study liver microcirculation. Therefore, unlike DEI, this method is unable to reveal the entire vascular tree of the liver lobe.

To explain the increased vascular density in cirrhotic livers, we carefully studied histopathological sections corresponding to the DEI images. We determined that the following factors caused the hypervascular changes during fibrosis progression. CCl_4_ toxification induces necrosis of hepatocytes around central venules. Extinction of liver parenchyma leads to approximation of afferent (portal) and efferent (hepatic) venules [26]. Loss of hepatocytes and proliferation of fibrotic tissue change the original ratio between liver parenchyma and mesenchyma, increasing the density of vessels. Up-regulation of factors related to vascular development, i.e. vascular endothelial growth factor, angiopoietin-1 and platelet-derived growth factor (see Figure S2) drives vascularization or angiogenesis in diseased livers. We also confirmed that the newly developed vessels were portal venules and sinusoidal vascularizations (afferents) instead of hepatic venules (efferents).

One outstanding limitation of this two dimensional DEI technique adopted in the study is that surface vessels may obscure the internal vascular structure of the liver, which hindering differentiation afferent from efferent vessels within the entire liver lobe. To overcome this, we are currently evaluating three-dimensional DEI as a diagnostic tool. Three-dimensional DEI will be able to display all vascular structures; we presume that this method will provide more information. Besides three-dimensional DEI, in vivo DEI is the new direction of this image modality. To date, the DEI technique has been used to study joints, cartilage, brain and lungs in vivo [27]–[31]. DEI has higher sensitivity and specificity in the diagnosis of early breast cancer than traditional mammography [32]. However, most assessments of hepatic microvessels by DEI used ex vivo liver samples owing to the deep location, thickness and movement of the liver during respiration. These difficulties may be overcome by incremental changes in X-ray flux and more efficient CCD to increase the acquisition speed to video rates. In addition, large size beams compatible with the size of the animal are needed for in vivo applications. Despite these challenges, in vitro DEI may be able to visualize microvascular changes corresponding with cirrhosis, thus improving our understanding of the pathogenesis of cirrhosis. Moreover, it may aid in clinical decision-making by allowing real-time images that can clearly illustrate the stages of cirrhosis from a microvascular perspective.

In conclusion, synchrotron-based DEI is compatible with radiological and pathological analysis. Our results are consistent with previous reports that angiogenesis is directly proportional to fibrosis progression, and further reveal that the microvascular changes accompanying cirrhosis cannot fully reverse during fibrosis regression. DEI has the potential to be a new tool for assessing the prognosis of cirrhosis by the severity of microvascular abnormalities during various stages of the disease.

## Supporting Information

Figure S1 **Portal vein (red arrow), bile duct (red arrowhead) and central vein (yellow arrow) were illustrated by bile duct ligation rat models.**
(TIF)

Figure S2 **Quantitative analysis of angiogenic related factors**. VEGF, Ang-1, PDGF mRNA (A) using real time polymerase chain reaction were showed**.** Enzyme-linked immunosorbent assay quantification of VEGF protein level (B). Change of angiogenic factors was parallel to the extent of vascular remodeling and severity of cirrhosis. Results are expressed the mean ±SEM. * *p*<0.05 compared with the control group. # *p* <0.05 compared with the cirrhotic group.
(TIF)
