# Supplementary figures and images for: A New Conversation between Radiology and Pathology-Identifying Microvascular Architecture in Stages of Cirrhosis via Diffraction Enhanced Imaging In Vitro
Source: PLoS One. 2014 Feb 4;9(2):e87957. doi: 10.1371/journal.pone.0087957 (PMC3913676; doi:10.1371/journal.pone.0087957)

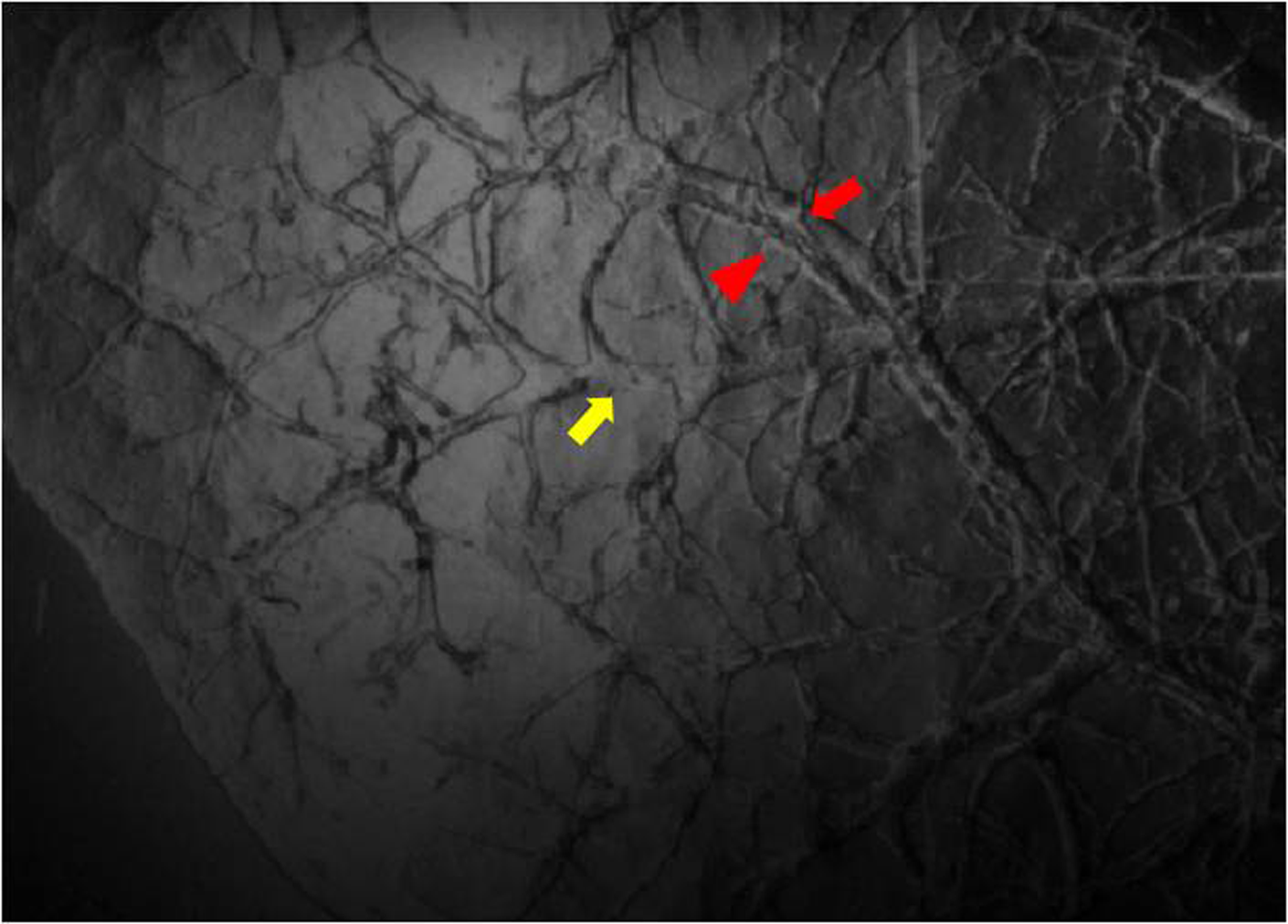

Supplement: Figure S1 — Portal vein (red arrow), bile duct (red arrowhead) and central vein (yellow arrow) were illustrated by bile duct ligation rat models. (TIF) [file pone.0087957.s001.tif]

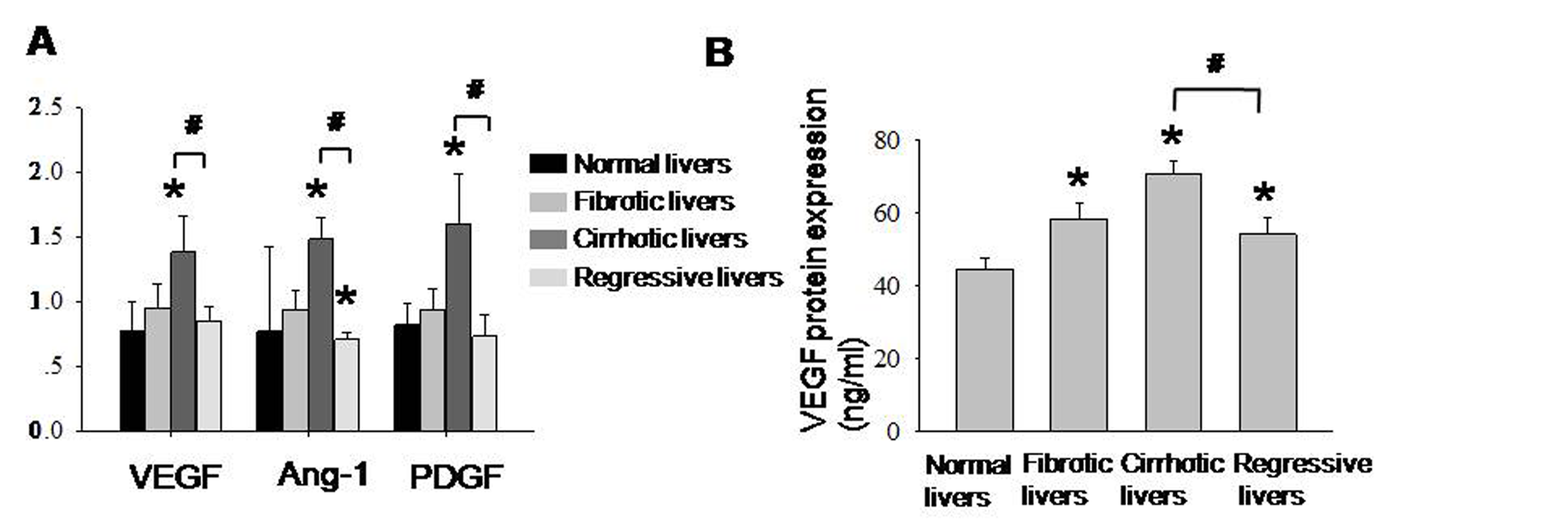

Supplement: Figure S2 — Quantitative analysis of angiogenic related factors. VEGF, Ang-1, PDGF mRNA (A) using real time polymerase chain reaction were showed. Enzyme-linked immunosorbent assay quantification of VEGF protein level (B). Change of angiogenic factors was parallel to the extent of vascular remodeling and severity of cirrhosis. Results are expressed the mean ±SEM. * p<0.05 compared with the control group. # p <0.05 compared with the cirrhotic group. (TIF) [file pone.0087957.s002.tif]
